# Tolerizing Strategies for the Treatment of Autoimmune Diseases: From *ex vivo* to *in vivo* Strategies

**DOI:** 10.3389/fimmu.2020.00674

**Published:** 2020-05-14

**Authors:** Anje Cauwels, Jan Tavernier

**Affiliations:** ^1^VIB-UGent Center for Medical Biotechnology, Ghent University, Ghent, Belgium; ^2^Orionis Biosciences, Ghent, Belgium

**Keywords:** autoimmunity, dendritic cells, tolerance, type-I-IFN, tolerogenic dendritic cells, pDC, cDC

## Abstract

Autoimmune diseases such as multiple sclerosis (MS), type I diabetes (T1D), inflammatory bowel diseases (IBD), and rheumatoid arthritis (RA) are chronic, incurable, incapacitating and at times even lethal conditions. Worldwide, millions of people are affected, predominantly women, and their number is steadily increasing. Currently, autoimmune patients require lifelong immunosuppressive therapy, often accompanied by severe adverse side effects and risks. Targeting the fundamental cause of autoimmunity, which is the loss of tolerance to self- or innocuous antigens, may be achieved via various mechanisms. Recently, tolerance-inducing cellular therapies, such as tolerogenic dendritic cells (tolDCs) and regulatory T cells (Tregs), have gained considerable interest. Their safety has already been evaluated in patients with MS, arthritis, T1D, and Crohn’s disease, and clinical trials are underway to confirm their safety and therapeutic potential. Cell-based therapies are inevitably expensive and time-consuming, requiring laborious *ex vivo* manufacturing. Therefore, direct *in vivo* targeting of tolerogenic cell types offers an attractive alternative, and several strategies are being explored. Type I IFN was the first disease-modifying therapy approved for MS patients, and approaches to endogenously induce IFN in autoimmune diseases are being pursued vigorously. We here review and discuss tolerogenic cellular therapies and targeted *in vivo* tolerance approaches and propose a novel strategy for cell-specific delivery of type I IFN signaling to a cell type of choice.

## Tolerance-Inducing Cells

Dendritic cells (DC) are best known for their antigen (Ag) processing and presenting functions, driving immunological responses directed against pathogens and malignant cells. Nevertheless, they are also crucial for coordinating immunological tolerance and preventing autoimmunity. Several types of DCs exist: conventional (cDC), plasmacytoid (pDC), and monocyte-derived (moDC). They all originate from CD34^+^ hematopoietic progenitor cells in the bone marrow. For a long time, it was generally believed that differentiation via macrophage/DC progenitors (MDC) gave rise to either the monocyte/macrophage lineage or to common DC progenitors (CDP), which further differentiated into either pDCs or pre-cDCs (1, 2). Recently, however, single-cell analysis formally demonstrated that pDCs do not develop from myeloid but from lymphoid progenitors, indicating an early divergence of pDC and myeloid-derived cDC lineages (3). Monocyte-derived DCs (moDCs) differentiate from monocytes during inflammation, induced by cytokines such as GM-CSF, IL-4, and TNF.

Vaccination with or induction of tolerogenic DCs (tolDC) could constitute a powerful therapy for autoimmune diseases. As many studies do not separate cDCs from moDCs in their analysis, it is not unequivocally clear whether endogenous moDCs also contribute to immune tolerance, besides cDCs and pDCs (4). In humans, DC research and experimental therapy by necessity focuses on moDCs, generated *ex vivo* by cytokine treatment of peripheral blood monocytes obtained via leukapheresis. To what extent these artificially produced moDCs really resemble primary endogenous DCs is not clear. It has been shown that they share some functional features with cDCs, but their overall gene expression patterns are much closer to monocytes than to any DC subset (2).

In mice, pDCs have been identified to be crucial for tolerance in several autoimmune disease models. Although most cells in the body are able to produce type I interferon (IFN-I), pDCs have been termed natural IFN-I-producing cells because of their unique adaptations to nucleic acid-sensing, which result in rapid and robust IFN-I release. Nevertheless, their *in vivo* contribution to antiviral and other infectious immune responses is probably less crucial than originally assumed (5). In Experimental Autoimmune Encephalomyelitis (EAE, the mouse model for MS), αPDCA1-induced pDC depletion or selective abrogation of MHCII expression on pDCs exacerbates EAE from the onset on (6, 7), while cDC depletion in cDC11-iDTR mice worsens disease during the later effector phase (8). In addition, PDCA1^+^ or SiglecH^+^ CD11c^*int*^ pDCs differentiated *ex vivo* from bone marrow-derived cells induce recovery (9). Also in acute graft-versus-host-disease (GvHD, induced via allogeneic bone marrow transplantation) and cardiac allograft models (10, 11), as well as in RA, asthma, T1D, and even atherosclerosis (12–15), pDCs have well-demonstrated tolerogenic functions, predominantly dependent on IDO (indoleamine-2,3-dioxygenase) and resulting in Treg induction and expansion (2, 4, 16).

In addition, type 1 and/or type 2 conventional DCs (CD8^+^ DEC205^+^ cDC1, C11b^+^ DCIR2^+^ cDC2) may also contribute to peripheral Treg differentiation and/or expansion and hence tolerance, both in homeostasis (17) and in certain autoimmune diseases such as EAE (4, 18–20). Also, in GvHD, host CD11c^+^ cDCs were shown not to be required for the induction of disease but rather to restrict alloreactive T cell expansion (21). In addition, protection against GvHD was recently revealed to involve the tolerogenic action of both CD8^+^ cDC1 and CD11b^+^ cDC2 (22, 23). In T1D, however, there is preclinical evidence for a predominant tolerogenic role for DCIR2^+^ cDC2, driving Treg expansion rather than differentiation (2, 24).

The mechanism by which tolDC instigate tolerance clearly involves the induction and expansion of Tregs. These are CD4^+^ Foxp3^+^ and may be generated in the thymus as natural Tregs or induced in the periphery as iTregs. Tregs are known to exert their immunosuppressive effect mainly via IL-10 and TGFβ production, which have well-established inhibitory effects on effector T cells (Teff) and positive effects on regulatory B cells (Bregs). Furthermore, Tregs may spread peripheral tolerance by generating tolDC from DC progenitors or by maintaining cDCs in an immature state (25–28). While most studies have reported no differences in the numbers of circulating Tregs in MS, RA, or T1D patients, defects in Treg phenotype and suppressive and migratory capacity have been demonstrated (29–32). Bregs represent a small population of B lymphocytes participating in immune suppression. Many of the different B cells with suppressive characteristics are CD5^+^ (33). A particular population, which is CD5^+^ CD1d^+^, are very potent producers of IL-10 and are hence often referred to as B10 lymphocytes. Like Tregs, Bregs perform their regulatory functions primarily via the production of IL-10 and TGFβ as well as IL-35 (34). They have recently been recognized as very important immune modulators in various autoimmune diseases, including MS, RA, T1D, and IBD, offering novel potential strategies for therapeutic interventions (35–39).

## *Ex Vivo* Tolerance-Inducing Cellular Therapies in Clinical Trials

The number of patients suffering from autoimmune diseases and allergies is rising dramatically (40). To avoid or dampen the aberrant harmful immune response against a specific (auto)Ag, immunological tolerance is warranted. Dampening of the immune response is also required for people receiving organ or stem cell transplants. This is currently achieved by administering “all-purpose” immunosuppressive drugs, which cause both immediate and late side effects, including increased risk for life-threatening infections and malignancies.

With the identification of tolerance-inducing cell types, significant progress has recently been made in the manufacturing and usage of tolerance-inducing cells. However, as these autologous cells are generated and manipulated *ex vivo*, this personalized therapy is very laborious and expensive, with many challenges, pitfalls, and safety issues (41, 42). In addition, it remains unclear whether these artificially engineered cells adequately resemble their endogenous primary counterparts *in vivo*.

**FIGURE 1:**
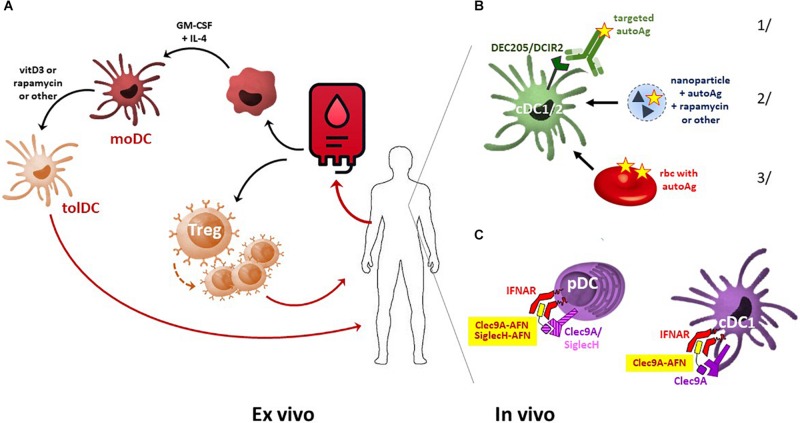
Comparison of *ex vivo-* and *in vivo*-generated tolDC. **(A)** For cellular tolDC therapy, monocytes are isolated from patient-derived peripheral blood, driven into moDC development using cytokine therapy, and subsequently tolerized by immunosuppressive agents such as vitamin D3 or rapamycin. These autologous tolDCs are then used for patient-specific treatment. From peripheral blood, Tregs may also be sorted and further expanded *ex vivo*. Once injected back into the patient, these Tregs dampen the immune system via multiple pathways, including the suppression of DC maturation. **(B)** *In vivo* induction of tolDC may be achieved by several approaches. Examples include delivering autoAg to DCs specifically via 1/antibody-mediated targeting of DC surface markers, 2/encapsulation in nanoparticles, microparticles, or liposomes, loaded (or not) with an immunosuppressive agent, or 3/infusion of Ag-carrying erythrocytes that will be cleared via phagocytosis predominantly by DCs and macrophages. **(C)** Delivery of self-Ag may add to disease development in a pro-inflammatory microenvironment, and autoAg patterns are not always uniform or stable over time. Alternatively, selective delivery of IFN-I signaling in pDC and cDC1 by AcTaferons (AFNs, targeted using SiglecH or Clec9A single-domain antibodies) may safely and cell-specifically induce systemic tolerance.

Amongst the different tolerogenic cell types, the application of tolDC is most advanced (Figure 1A). The first clinical study on tolDC therapy was performed in 2011 in adult T1D patients. Since then, phase I and II clinical trials have been conducted for T1D, RA, Crohn’s disease, and MS. TolDC therapy is safe and shows signs of causing clinical improvement in certain patients (43, 44). In addition, tolDCs have also proven their immune dampening and thus protective potential in animal models of transplantation and allergic asthma, and clinical trials in kidney and liver transplant recipients are being set up (45–47). Once injected, tolDCs are expected to induce tolerance through various mechanisms, including the induction of Tregs and Bregs, and the stimulation of autoreactive T cell anergy and apoptosis (43).

Not only tolDC but also other myeloid regulatory/immunosuppressive cell types are currently being explored, including immature myeloid-derived suppressor cells (MDSC) and activation-induced regulatory macrophages (Mregs) (48, 49). The latter are monocytes matured through adherence to plastic surfaces and exposure to various serum factors and/or cytokines and acting through IDO, IL-10, and TGFβ. *In vitro*, human Mregs are capable of deleting activated T cells, suppressing T-cell proliferation, and driving naive T cells to become Tregs, and the protective capacity of donor-derived Mregs is being explored in kidney transplant recipients (50).

The *ex vivo* expansion of autologous blood-derived Tregs has also been a clinical focus for inducing tolerance in autoimmune diseases such as GvHD, T1D, MS, Crohn’s disease, SLE, autoimmune hepatitis and uveitis, and in kidney transplant patients (43, 47). The outcomes of the completed trials indicated that Treg therapy is feasible and safe. However, like tolDC generation, this strategy requires personalized, complex, and expensive manufacturing processes. In addition, current techniques lack specificity as they expand polyclonal rather than Ag-specific Tregs and also carry the risk of expanding so-called unstable Tregs that may lose their tolerogenic function and undergo transformation to pathogenic T cells, exacerbating disease.

Still another cell type with tolerogenic capacity is the mesenchymal stromal cell (MSC) population, a non- hematopoietic, multipotent, and self-renewing population found in bone marrow as well as in other tissues such as umbilical cord, muscle, and adipose tissue, that has a proven potential to modulate anti-inflammatory monocytes and macrophages, DCs, B and T lymphocytes, and NK cells (51, 52). Clinical trials with *ex vivo-*expanded MSC have been successfully conducted, showing good tolerability and therapeutic potential in MS, RA, Crohn’s disease, SLE, and GvHD. A significant advantage of MSC therapy over other cell-based tolerogenic therapies is their lack of MHC expression, expanding the source of cells from autologous to allogeneic. In addition, MSC sources are multiple, including umbilical cord tissue and lipo-aspirate (43).

## Generation and Mechanisms of tolDC

Human autologous tolDCs are generated *ex vivo*, starting from peripheral blood monocytes obtained via leukapheresis and cell sorting (Figure 1A). After culturing in the presence of GM-CSF and IL-4 to drive their development into moDCs, tolerization is usually achieved by treating with immunomodulatory agents such as vitamin D3, rapamycin, dexamethasone, corticosteroids, or specific cytokines (IL-10, TGFβ, IFNβ) (Figure 1A). Depending on the nature of the tolerizing protocol, the exact mechanisms involved in inducing systemic tolerance may diverge (53). Interestingly, whatever the tolerization protocol, this *ex vivo* approach will automatically lead to the generation of moDCs, which have gene expression patterns closer to monocytes than to DCs (2). As already mentioned, the endogenous DC subset that is typically found to be involved and necessary for protection in various autoimmune diseases is primarily the pDCs. In view of the recent finding that pDCs are not myeloid-derived, as was thought for decades, but are rather lymphoid-derived (3), the efficacy/efficiency of myeloid-derived moDCs as tolDCs can be questioned. Also in the cancer immunotherapy field, the disappointing performance of moDCs has been suggested to be due to an intrinsic lack of biological potency as compared to endogenous cDCs (54).

Both pDCs and cDCs induce tolerance by promoting immunosuppressive Treg differentiation and function. Important endogenous signaling agents for these processes include IL-10, TGFβ, retinoic acid (RA), and kynurenine produced by IDO (55). IDO is not expressed constitutively in DCs and requires induction by various pro- and anti-inflammatory mediators such as IFNs and TGFβ. Tolerance induced by IDO may even result in so-called “infectious” tolerance, spreading from one cell to another due to kynurenines acting as activating ligands for the aryl hydrocarbon receptor (AhR) and as such for the induction of IDO expression in other cells (56). In addition, IDO activity results in tryptophan catabolism and hence metabolic stress, negatively affecting Teff proliferation and survival. Furthermore, pDCs and cDCs can also induce peripheral tolerance by inducing Teff cell anergy, i.e. functional inactivation due to checkpoint inhibitions (18).

## Type I Interferon in Autoimmune Diseases

At least 80 different forms of autoimmune diseases exist. Together, approximately 8% of the world’s population suffers from an autoimmune disease, and prevalence is sharply increasing (40). Autoimmune diseases mainly afflict women (>80%), strike at the prime of life, and cause significant debilitation, morbidity, and even mortality. In many of the most prevalent autoimmune diseases, various roles for type I IFN (IFN-I) have been described. Type I IFNs consist of a large group of structurally similar cytokines and include 13–14 subtypes of IFNα along with IFNβ, IFNε, IFNκ, IFNω, IFNδ, IFNζ, and IFNτ, all signaling through the same receptor composed of two subunits, IFNAR1 and IFNAR2. In most autoimmune models, both pathogenic and protective roles have been described, primarily for IFNα and/or IFNβ, probably depending on the disease state and the microenvironment. In general, it is important to realize that cytokines such as IFNα and IFNβ may exert different functions depending on the inflammatory context, location, and activation status of the responsive cell types.

IFNβ was the first disease-modifying therapy approved for the treatment of MS patients. Despite its therapeutic use for more than a quarter of a century, its precise mode(s) of action and specific target cells are still not completely understood. Most MS patients benefit from IFNβ therapy, but some exhibit no response or even a worsening (57). This may be due to differential effects on different cell types. In addition, side effects due to systemic toxicity preclude dose escalation and trigger therapy drop out.

In mice, triggering endogenous IFN-I release via TLR therapies can protect against IBD induced by DSS or IL-10 deficiency (58–60). Next to the activation of TLR7 or TLR9, endogenous IFN-I may also be induced by the ER-associated protein STING (stimulator of IFN genes), activated by cyclic dinucleotides. STING was shown to be important for maintaining intestinal homeostasis, and it was hence proposed that modulating the STING pathway may be of benefit in IBD (61). However, endogenous STING signaling induces both pro- and anti-inflammatory cytokines, and indeed, STING agonists were recently shown to exacerbate colitis (62). Collectively, these reports suggest that the beneficial effect of IFN-I in IBD is probably local and/or cell-specific.

From experiments performed in diabetic mice and rats, the role of IFN-I in T1D pathogenesis was originally believed to be beneficial (16). Later, this protective role was questioned, as IFNα produced by pancreatic β-cells or by pDC was shown to hasten murine diabetes progression (63, 64), and a detrimental role for pDC-derived IFNα in the initiation of T1D was eventually concluded from experiments in NOD mice (65). Nevertheless, ingestion of low-dose IFNα preserved β-cell function in recent-onset T1D patients (66), and additional clinical trials have since shown protective effects of ingested IFNα in patients suffering from MS (67).

Also in arthritis models and human RA, various roles for IFN-I have been proposed, ranging from detrimental to protective. Several experiments performed in both mice and monkeys, as well as pilot studies in RA patients, clearly suggest clinically meaningful improvement due to IFNβ treatment (68). Interestingly, a protective role has also been demonstrated for pDC, and clinical trials with tolDC are ongoing (47, 69). The controversial results using systemic IFN-I could possibly indicate local and/or cell-specific differential effects of IFN-I.

Using a murine GvHD model, TLR7 agonists were found to protect IFNAR1-dependently, involving the tolerogenic action of cDCs and increased Treg responses (22). Furthermore, selective activation of IFN-I pathways prior to hematopoietic stem cell transplantation was shown to be dependent on IFN-I signaling in CD11c^+^ DC, reducing their ability to stimulate allogeneic T cells (23).

In addition, it has been suggested that the lack of IFN-I secretion by pDCs contributes to the development of a TH2 response in allergic asthma and that treatment of chronic allergic diseases with IFN-I may be a promising way to induce tolerance (70).

## Strategies to Induce tolDC *In Vivo*

*Ex vivo-*generated tolDCs are well-tolerized and may have protective effects, but they also have several disadvantages, as they represent a personalized, laborious, and expensive therapy that raises many safety and economic concerns. To overcome these limitations, new approaches to induce tolDCs *in vivo* are being vigorously explored (Table 1). Examples include selective self-Ag targeting toward the DC receptor DEC205 before or after EAE immunization (19, 71, 72), or toward the pDC receptor SiglecH or the cDC2 receptor DCIR2 before EAE immunization, to promote immunological tolerance (20, 71, 73) (Figure 1B). Other successful approaches include injection of autoAg-containing nano- or microparticles or liposomes. These are taken up via phagocytosis or endocytosis, predominantly by antigen-presenting cells (APC, including DCs and myeloid cells), and disease-relevant peptides or proteins can be co-encapsulated with immunosuppressive agents such as rapamycin, IL-10, NFκB inhibitors, or AhR ligands (74–79). Most of these reported studies were performed in EAE and T1D models, but their efficacy has also been illustrated in other autoimmune diseases such as arthritis and IBD (76, 80, 81) and in various transplantation models (82) (Table 1). In addition, transfusion with autoAg-decorated red blood cells (rbc) (Figure 1B), which are known to be preferentially phagocytosed by DCs and macrophages, has recently proven its efficacy in both EAE and NOD diabetic mice (83). Importantly, maturation or activation signals for DCs, present under inflammatory conditions, may abrogate the tolerogenic protection conveyed by autoAg delivery (20), and as such endogenous inflammation could turn a self-Ag-based DC tolerizing therapy into one further exacerbating disease (44). In addition, self-Ag patterns are not always uniform or stable during disease development and progression.

**TABLE 1:** Summary of approaches to inducing tolDC *in vivo.*

**Experimental mode**	**Tolerizing approach**	**Cells targeted**	**Specificity**	**Timing**	**Results**	**References**
EAE (PLP, SJL)	PLP Ag delivery	cDC1 + LC	DEC205 Ab	−10 d/−15 d	Prevent and reduce disease severity ∼prevent Ag response + T suppressive mechanism	[72]
EAE (MOG, C57Bl/6)	MOG Ag delivery	pDC	SiglecH Ab	−1 d	Delay onset and reduce disease ∼prevent Ag response, no effect Tregs	[73]
EAE (MOG, C57Bl/6)	NP + peptide + AhR ligand	APC	Non-specific	d0	Suppress disease progression ∼expand Tregs	[79]
EAE (MOG, C57Bl/6)	MOG Ag delivery	cDC1 + LC cDC1 migratory cDC1 resident cDC2	DEC205 Ab Langerin Ab Treml4 Ab DCIR2 Ab	−14 d −14 d −14 d −14 d	Prevent and reduce disease Prevent and reduce disease Prevention/reduction minimal Prevention/reduction minimal ∼Protection correlates with Treg generation	[71]
EAE (spinal cord, C57Bl/6)	MOG Ag delivery	cDC1 + LC	DEC205 scFv	−7 and −3 d or +7 and 11 d	Prevent disease TGFβ-dependently ∼reduce IL-17 & IFNγ in CD4^+^ T cells ∼induce TGFβ^+^ capacity in DCs	[19]
EAE (MOG, C57Bl/6)	NP + peptide + IL-10	APC	Non-specific	−30 and −15d or +8 and 22d	Reduce disease severity ∼reduced IFNγ and IL-17 by splenic T cells	[77]
EAE (PLP, SJL)	Highly negative MP	Inflammatory mono	via MARCO	+7 d; start relapse	Prevent disease; relapse ∼reduced inflammation CNS (especially DC)	[81]
EAE (MOG, C57Bl/6; MPB, B10.PL)	NP + Ag	Liver sinus EC accumulation	Selected NP	+1 d; +8−12 d	Prevent; reduce disease score ∼TGFβ and Treg dependently	[90]
EAE (PLP, SJL)	NP + Ag + rapamycin	APC	Non-specific	−14 and −21 d; +13 d	Prevent disease; relapse ∼prevent Ag response, induce Treg/Breg	[78]
EAE (MOG, PLP, C57Bl/6)	NP + MHCII-MOG-peptide or MHCII-PLP-peptide	APC	Non-specific	+14 d; +21 d	Reduce disease severity ∼Ag-ex perienced Teff→Tr1 (APC-dependent) ∼formation and expansion Bregs	[80]
EAE (MOG, C57Bl/6)	MOG Ag decorated rbc	Phagocytes	Non-specific	7 d/+5 d/+11 d	Prevent or cure disease ∼Th17 decrease in CNS	[83]
EAE (PLP, SJL)	NP + peptide + rapamycin	APC	Non-specific	+14 d = peak	Prevent relapse ∼prevent Ag response + expand Tregs	[74]
EAE (PLP, SJL)	PLP Ag delivery	CD11b^+^ cDC2	DCIR2 Ab	−10d	Prevent and reduce disease ∼reduce pathogenic T_*H*_ + expand Tregs	[20]
EAE (MOG, C57Bl/6)	Engineered IFNα delivery	pDC, cDC1	SiglecH sdAb or Clec9A sdAb	+7 d/+12 d	Delay onset, reduce disease progression ∼IDO/TGFβ^+^ pDC; IL-10/TGFβ^+^ Treg and Bregs	[89]
Diabetes (p31-T transfer)	p31 peptide delivery	rbc → uptake DC	Ly76 scFv → non-specific	+8 h till 7 d	Prevent hyperglycemia ∼deletion transferred diabetogenic T cells	[91]
Diabetes (NOD/BDC2.5)	NP + IAg7 (NOD/BDC2.5 MHCII)	APC	Non-specific	10 wk of age	Prevent incidence ∼Ag-experienced Teff → Tr1 (APC-dependent) ∼formation and expansion Bregs	[80]
Diabetes (NOD+BDC2.5-T)	BDC2.5 peptide delivery	cDC2 cDC1	DCIR2 Ab DEC205 Ab	−1 and 0 d −1 and 0 d	Delay diabetes induction ∼ T cell apoptosis no effect	[24]
Diabetes (NOD mice)	NP + Ins Ag + AhR ligand	APC	Non-specific	8 wk of age	Reduce disease development ∼tolDC, Treg differentiation	[92]
Diabetes (NOD mice)	Ins Ag decorated rbc	Phagocytes	Non-specific	10 wk of age	80% protection	[83]
IBD (DSS, C57Bl/6)	Highly negative MP	Inflammatory mono	via MARCO	+1−6 d	Suppress disease score ∼reduced inflammation/colon	[81]
Inflammatory arthritis	liposomes + Ag + NFκB⊥	cDC, pDC, mf	Non-specific	+6 d	Reduce disease severity ∼induction Tregs, suppression Teffs	[76]
Arthritis (CIA)	NP + MHCII-collagen peptide	APC	Non-specific	@130% swelling	Reduce disease severity ∼Ag-experienced Teff → Tr1 (APC-dependent) ∼formation and expansion Bregs	[80]
Skin transplantation	MHC-I monomer delivery	cDC2	DCIR2 Ab	−14 d	Long term allograft survival if CD8-depleted	[93]
Liver transplantation	NP + tacrolimus	APC	Non-specific	+4 till 10 d	Prolong allograft survival	[94]
Heart transplantation	Targeted NP + αCD3	HEV, LN	MECA79 Ab	−1 d till 3 d	Prolong allograft survival ∼Treg dependent	[95]
Heart transplantation	HDL-NP + rapamycin	Myeloid cells (mf)	Non-specific	+6 d	Long term allograft survival ∼Mreg dependent	[96]

*Ab, antibody; Ag, antigen; AhR, Aryl hydrocarbon receptor; APC, antigen-presenting cell; cDC, conventional dendritic cell; d, day; EC, endothelial cell; HEV, high endothelial venules; Ins, insulin; LC, Langerhans cell; LN, lymph node; mf, macrophages; MOG, myelin oligodendrocyte glycoprotein; mono, monocytes; MP, microparticles; NP, nanoparticles; pDC, plasmacytoid dendritic cell; PLP, proteolipid protein; rbc, red blood cells; sdAb, single-domain antibody; scFv, single-chain variable fragment; wk, week.*

Not only DCs and myeloid cells are being targeted; efforts are also underway to selectively stimulate the *in vivo* expansion of Tregs. So far, most trials have concentrated on the use of low-dose IL-2 to achieve this, since IL-2 is crucial for T-cell proliferation and its receptor is most abundant on natural Tregs, but an optimal and long-lasting regime has not yet been found and agreed upon (30, 43). Furthermore, no pharmacological approaches are currently available to selectively expand autoAg- or disease-specific Tregs *in vivo*.

**FIGURE 2:**
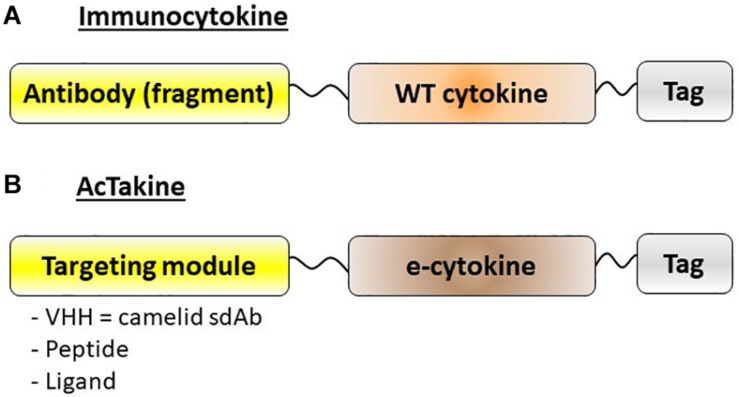
Schematic representation of immunocytokines and AcTakines. **(A)** Immunocytokines are typically engineered by coupling a wild-type (WT) cytokine to a targeting module, usually an antibody or antibody fragment. **(B)** AcTakines consist of a mutated (engineered) e-cytokine with reduced cognate receptor affinity, coupled C-terminally via a 20xGGS linker to a targeting moiety. In general, a camelid-derived single domain antibody (sdAb = VHH) is used for the latter, although peptides or ligands can also be employed. For purification purposes, AcTakines are decorated with a C-terminal affinity tag.

Given the possible protective role of IFN-I in autoimmune diseases, especially in MS, we decided to apply our targeted AcTaferons in the EAE model. AcTaferons (AFNs) are IFN-I based AcTakines (Activity-on-Target cytokines). Basically, AcTakines are a novel class of engineered immunocytokines, the key difference between AcTakines and immunocytokines being the exclusive use of mutant (engineered) e-cytokines with severely reduced receptor affinity instead of wild-type (WT) cytokines (84) (Figure 2). While immunocytokines, where WT cytokines are fused to targeting antibodies or antibody fragments, can still bind with great affinity to their ubiquitous receptors while traveling through the body, causing residual side effects and their systemic removal [the so-called “sink” effect (85)], AcTakines cannot signal when administered systemically except in those cells that express a surface molecule specifically recognized by the targeting moiety linked to the mutant cytokine. As a result, AcTakines do not cause the multiple toxic side effects usually accompanying cytokine therapies. In addition, they provide unique research tools for dissecting the *in vivo* cell-specific functions of pleiotropic cytokines under normal or pathophysiological conditions. Thanks to a convenient “plug-and-play” assembly of modular building blocks, AcTakines can be engineered easily by coupling various mono- or multimeric e-cytokines to targeting moieties such as camelid-derived single-domain antibodies (sdAb, VHH), peptide motifs specifically recognized by receptor isoforms, or ligands interacting with their cell-specific cognate receptors. During recent years, we have successfully and safely employed various cell-specific AcTakines as potential anti-tumor therapies (86–88). Recently, we also obtained evidence in EAE that DC-targeted AFN can be used to specifically target IFN-I signaling to DCs as an *in vivo* method to induce tolerance (89) (Figure 1C). Systemic tolerance was evident in pDCs (increased numbers and an enhanced tolerogenic signature including IDO and TGFβ production) as well as in Tregs and Bregs, both of which produced significantly more IL-10 and TGFβ. Interestingly, pDC targeting was superior to cDC1 targeting during early progression of EAE, but cDC1 targeting later during disease progression significantly added to the protection. In contrast to therapy with autologous *ex vivo*-generated moDCs derived from a cell lineage that may not be optimal (myeloid), AFNs deliver the IFN-I signaling potential specifically to endogenous pDCs and cDC1s *in vivo*. Furthermore, cell-specific targeting not only limits the possibility of aspecific toxic side effects but also avoids signaling in unwanted cell types. The relevance of the latter becomes clear when comparing the protective capacities of untargeted WT IFN-I with CD8- or DC-targeted AFN. While WT IFN-I can delay disease onset and DC-targeted AFN provides profound protection, CD8-targeted AFN worsens disease (89). This strategy still leaves many more options open, such as selective targeting to B lymphocytes, specific myeloid subsets, and more.

## Author Contributions

All authors listed have made a substantial, direct and intellectual contribution to the work, and approved it for publication.

## Conflict of Interest

JT was employed by company Orionis Biosciences, who provided funding for the study. The remaining author declares that the research was conducted in the absence of any commercial or financial relationships that could be construed as a potential conflict of interest.
